# Evidence of Genetic Isolation and Differentiation Among Historically Fragmented British Populations of Common Juniper, *Juniperus communis* L.

**DOI:** 10.1002/ece3.71818

**Published:** 2025-07-20

**Authors:** J. Baker, J. Cottrell, R. Ennos, A. Perry, S. A'Hara, S. Green, S. Cavers

**Affiliations:** ^1^ UK Centre for Ecology & Hydrology Penicuik UK; ^2^ University of Edinburgh Edinburgh UK; ^3^ Forest Research Roslin UK

**Keywords:** genetic diversity, genetic isolation, habitat fragmentation, juniper, population genetics, SNP, SSR

## Abstract

Habitat fragmentation and population isolation pose a threat to the genetic diversity and adaptability of many species. The common juniper, *Juniperus communis* L., a keystone species for juniper scrub habitat and one of only three conifers that are native to the United Kingdom, has been in decline for more than a century in the United Kingdom and across its European range. Remnant UK juniper stands are now often small and highly fragmented, which has raised concerns for their resilience, especially in the face of climate change and the introduction of novel pathogens, such as *Phytophthora austrocedri.* This work presents a baseline genetic survey of native UK juniper populations and compares patterns of diversity between remnant stands and among three main population centres, or regions, in southern England, the Lake District, and Scotland, using both single nucleotide polymorphism (SNP) and simple sequence repeat (SSR) genetic markers. The aim was to evaluate the standing genetic diversity of native juniper stands, the impacts of habitat fragmentation, and to determine whether juniper populations are genetically isolated from one another. We found that juniper stands, while not completely isolated from one another, face substantial barriers to gene flow, especially between the three population centres. These centres also show different patterns of genetic diversity and population structure, indicating varying levels of internal gene flow. Our findings can provide a baseline from which to monitor the effectiveness of conservation activities, prioritize populations of concern, and guide genetic rescue efforts.

## Graphical abstract

The genetics of UK populations of common juniper were evaluated. Our findings suggest both limited gene flow among populations and evidence of genetic isolation. These findings can form a baseline to monitor these populations, identify priority populations, and guide genetic rescue efforts.

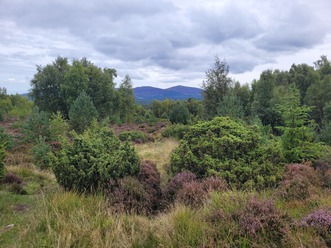

## 1 Introduction

Habitat fragmentation represents a threat to the genetic diversity of tree species as it can lead to a decline in gene flow between populations and a resultant increase in genetic drift and inbreeding in remnant populations (Aguilar et al. 2008; Dobeš et al. 2017; Young et al. 1996). The potential reduction of genetic diversity in fragmented populations may, in turn, impair the adaptive potential of those populations (Cavers and Cottrell 2015; Ennos 2015). Generally, features that facilitate the production of new genotypes, such as larger population size, inter‐population gene flow, and abundant natural regeneration, all maintain or increase the adaptive potential of that population. Therefore, conservation with the explicit goal of maintaining or increasing the genetic diversity of species and populations by promoting gene flow and natural selection, often called dynamic conservation, has become a recognized method with which to create more resilient forest populations (Cavers and Cottrell 2015; Fady et al. 2016; Finger et al. 2022; Hubert and Cottrell 2014; Lefèvre et al. 2013). The effects of habitat fragmentation vary by species and are determined by mating systems, life‐history traits, and the demographics of the meta‐population before the fragmentation occurred (Aguilar et al. 2008; Lowe et al. 2015). Neutral marker data have been widely used to evaluate the genetic consequences of fragmentation on tree populations and to manage their conservation and restoration (Cavers and Cottrell 2015; Dobeš et al. 2017; Ennos et al. 1998; Ennos 2015; Finger et al. 2022). Although some temperate tree species, such as Scots pine ( *Pinus sylvestris* ), have generally been found to have very high levels of gene flow among populations (Rodriguez 2019; Salmela 2011; Salmela et al. 2013), some species, such as yew ( *Taxus baccata* ), seem to be more sensitive to fragmentation (Chybicki et al. 2024). Here, we use a novel set of single nucleotide polymorphisms (SNPs) and a newly developed panel of simple sequence repeats (SSRs) to quantify the genetic diversity and infer the effects of habitat fragmentation on the keystone species common juniper, *Juniperus communis* L.

The common juniper has the widest global distribution of any conifer species, with a circumpolar range that extends from northern tundra in Russia and Canada as far south as the Mediterranean in Europe and the Central Rockies in North America (Thomas et al. 2007) It is one of only three conifers that are native to the United Kingdom. The species is morphologically variable and can grow as upright mid‐story trees, sprawling shrubs, or ground‐hugging stems (Carrer et al. 2019; Klimko et al. 2007; Knyazeva and Hantemirova 2020). It is dioecious, wind‐pollinated, and its seeds are primarily dispersed by birds (Adams and Thornburg 2010; García 2001; Surso 2018; Thomas et al. 2007). Individual junipers can live as long as 100–200 years, and most extant stands have a notable bias for mature or old individuals (Sullivan 2003; Thomas et al. 2007). Juniper trees are a keystone species for many of the communities in which they occur, providing habitats for lichens and bryophytes and abundant seasonal forage for animals. Furthermore, they can aid the recruitment of other tree species by acting as “nursery trees,” protecting young tree seedlings when they are particularly vulnerable to predation. Juniper also has a long history of human use, for example as a smokeless firewood (Thomas et al. 2007) and for both medical and culinary purposes (Al‐Snafi 2018).

Although juniper's considerable phenotypic variability and dispersal strategies (Hall 1990; Knyazeva and Hantemirova 2020; Thomas et al. 2007) might suggest a highly adaptable and resilient species, populations have been declining for at least the past century in both the United Kingdom and Europe. Consequently, it is listed as a priority species under the UK's Biodiversity Action Plan (McBride 2005), and many juniper communities are listed as Special Areas of Conservation under the EU Habitats Directive. In the United Kingdom, remnant juniper stands are generally small, and some, such as those in Southern England, are failing to regenerate naturally. Changes in land use, particularly grazing and the absence of regular natural disturbances (De Frenne et al. 2020; McBride 2005; Thomas et al. 2007), are considered the primary reasons for the lack of seedling recruitment, but there are many other factors that may contribute, including increasing temperatures (Gruwez, De Frenne, Vander Mijnsbrugge, et al. 2016; Verheyen et al. 2009) and changing soil nutrient compositions (Gruwez et al. 2014; Gruwez, De Frenne, Schrijver, et al. 2016; Pers‐Kamczyc et al. 2020, 2022; Verheyen et al. 2009). The lack of natural regeneration is resulting in populations that are both shrinking and aging, with more male‐biased sex ratios, which is especially concerning given that reproductive success may decrease as plants age (García et al. 1999; Ward 1982, 2007).

Previous genetic surveys of junipers in Western Europe have typically been restricted to relatively small geographic areas when compared to juniper's global range, and often differ in the genetic markers used, making direct comparisons difficult. However, one clear geographic pattern is that juniper populations on the British Isles tend to be more genetically distinct from one another than those on Mainland Europe (Michalczyk et al. 2010; Oostermeijer and De Knegt 2004; Provan et al. 2008; Reim et al. 2016; Vanden‐Broeck et al. 2011). Larger studies across Russia using a variety of methods and markers, including quantitative genetics and chloroplast DNA, have generally found a primarily east–west pattern of genetic differentiation, with evidence for a tertiary and quaternary genetic groupings in the North Caucasus and the Himalayas/southern Siberia (Hantemirova et al. 2012, 2017; Hantemirova and Bessonova 2023; Knyazeva and Hantemirova 2020). Previous studies have also found generally high degrees of genetic diversity within juniper populations, despite population fragmentation (Hantemirova et al. 2012, 2017; Hantemirova and Bessonova 2023; Michalczyk et al. 2010; Oostermeijer and De Knegt 2004; Provan et al. 2008; Reim et al. 2016; Vanden‐Broeck et al. 2011). Our study is the first to include samples from both Scotland and England and includes all of the subspecies that occur on the British Isles: *J. communis* spp. *communis*, *J. communis* spp. *nana*, and *J. communis* spp. *hemisphaerica* (hereafter abbreviated as *J. communis, Nana*, and *Hemi*, respectively). The three subspecies are primarily distinguished on the basis of differences in leaf morphology (Stace 2019), although *J. communis* and *Nana* may also be distinguished by their cone anatomy (Sullivan 2001). The three subspecies differ in their ranges, with *Nana* being restricted to the west coast of Scotland and Wales (G. Sullivan 2003; Thomas et al. 2007) and *Hemi* only present at a single site in Cornwall (Stace 2019; Thomas et al. 2007). Although the genetic differences among the subspecies are not clear, Sullivan (2001) found evidence that the prostrate growth habit of *Nana* was a genetic adaptation that was retained in a common garden trial, whereas prostrate *J. communis* cuttings demonstrated some phenotypic plasticity by adopting different growth habits when grown in a common garden trial. Sullivan (2001) did not, however, find support for their genetic distinction based on RAPD markers. Similarly, the genetic status of *Hemi* is unclear, and it is often regarded as an intermediate between the other two subspecies (Thomas et al. 2007).

The goal of this work was to provide a genetic survey that allows for the comparison of the larger Scottish populations with the smaller and more fragmented ones in the Lake District and southern England. Here, we use the term “populations” o refer to discrete stands of juniper trees, which in the United Kingdom are typically small and geographically fragmented, but acknowledge that some high‐latitude conifers, such as Scots pine, are capable of long‐distance gene dispersal and, therefore, form much larger meta‐populations (Beaton et al. 2022; Benavides et al. 2021; Cavers and Cottrell 2015; De Kort et al. 2013; Lefèvre et al. 2013; Young et al. 1996). This work uses both SNP and SSR genetic markers, as they are complementary approaches with different strengths and weaknesses (García et al. 2018). Quantifying the patterns of genetic diversity using these neutral genetic markers can inform researchers and conservationists about the population‐scale dynamics of gene flow, the effects of habitat fragmentation, and the development of effective management strategies. Our aim was to provide guidance to conservation managers in Britain and to target the selection of some British juniper populations as Gene Conservation Units (GCUs) under the European Forest Genetic Resources Programme (EUFORGEN).

## 2 Methods

### 2.1 Sampling Locations, Material Collection, DNA Extraction, and Marker Development

**FIGURE 1:**
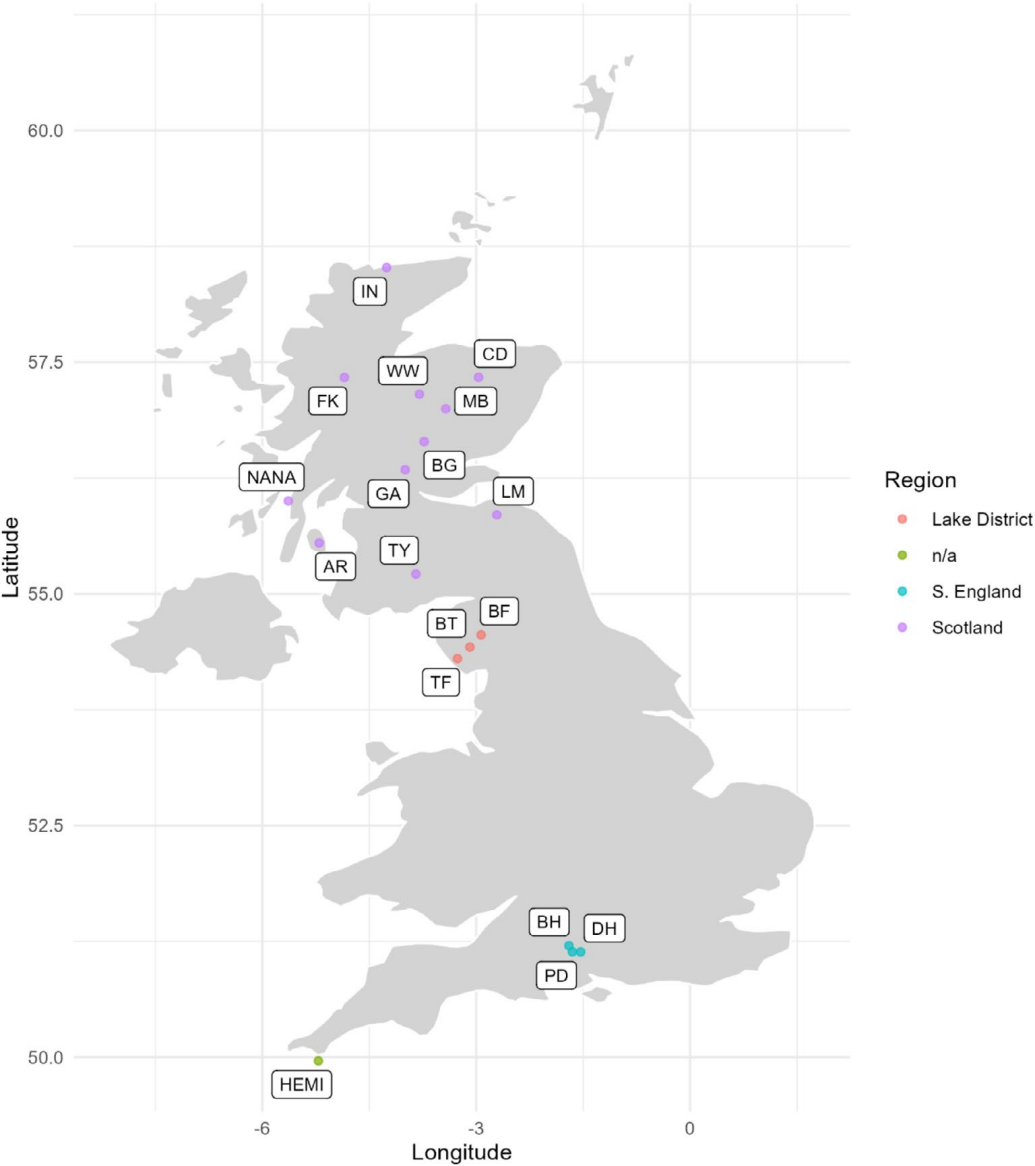
Locations of sampled populations, demarked with colored circles corresponding to each of the three regions; population name abbreviations given in Table 1.

Sixteen stands of *J. communis* and one each of *Nana* and *Hemi* were sampled from sites in England and Scotland and classified into the regions Scotland, Lake District, and Southern England, except for *Hemi*, which was an outgroup and excluded from these regional analyses (Table 1 and Figure 1). Needle samples were collected from mature juniper trees in 2019 and immediately stored at −20°C until processing.

**TABLE 1:** List of populations that were included in genetic analyses, including the abbreviations that are used in proceeding figures, the region that each population was assigned to, coordinates and the number (*N*) of individuals in both SNP and SSR datasets.

Population	Abbreviation	Region	Lat (°)	Long (°)	*N* for SNP	*N* for SSR
Gleann Dubh, Arran	AR	Scotland	55.55	−5.202	12	11
Blowick Fell	BF	Lake District	54.558	−2.931	10	10
Balnaguard Glen	BG	Scotland	56.644	−3.73	23	30
Bulford Hill	BH	S. England	51.204	−1.7	12	11
Blea Tarn	BT	Lake District	54.426	−3.088	10	8
Clashindarroch	CD	Scotland	57.338	−2.967	20	35
Danebury Hill	DH	S. England	51.137	−1.535	22	27
Fasnakyle	FK	Scotland	57.336	−4.849	22	26
Glen Artney	GA	Scotland	56.34	−3.996	23	24
Invernaver	IN	Scotland	58.521	−4.256	6	6
Lammermuir	LM	Scotland	55.853	−2.71	23	30
Morrone Birkwood	MB	Scotland	56.998	−3.426	7	6
Porton Down	PD	S. England	51.138	−1.652	22	24
Thwaites Fell	TF	Lake District	54.302	−3.263	22	25
Tynron	TY	Scotland	55.214	−3.846	15	15
Whitewell	WW	Scotland	57.155	−3.796	9	9
ssp. *nana*	*Nana*	Scotland	56.003	−5.635	8	
ssp. *hemisphaerica*	*Hemi*	Lizard Peninsula	49.961	−5.213	11	7

*Note:* The population “*Hemi*” is not included in regional analyses.

Prior to DNA extraction, 100 mg of needles were finely chopped using a razor blade, placed in a 2 mL microfuge tube with two 3 mm steel ball bearings, frozen in liquid nitrogen, and then ground using a Retsch MM 300 mixer mill at a frequency of 30/s for a total of 2 min. Razor blades and the glass cutting plate were cleaned with 70% ethanol between processing each sample. DNA was extracted for each sample using a Qiagen DNeasy Plant Pro kit (Qiagen, Germany) following the manufacturer's instructions. A SNP‐based assay was developed by the Plant Genomic Resources Centre (https://pgtb.fr/, Bordeaux, France). DNA from a panel of eight individuals from five sites (representing broad UK geographic coverage) was used to develop the markers following RAD‐Sequencing. Approximately 90,000 loci were initially detected by STACKS (Catchen et al. 2013), which were then filtered for the following criteria: (1) loci where a genotype was called for all individuals (including technical replicates; 9539 loci remaining), (2) loci with functional technical replicates (9374 loci remaining), (3) loci with unique SNPs in the returned sequence (807 loci remaining), (4) loci with at least 3 genotypes (198 loci remaining) and (5) loci where the unique SNP site is not in the first or last 20 bases of the sequence to allow for primer design (175 loci remaining). Of these 175 loci, 80 were selected for two multiplex Sequenom assays (Bradić et al. 2012), which ultimately provided data at 74 SNP loci for all samples (Table A1).

A new set of microsatellite markers was developed for this study (Table A2). DNA samples were sent to Microsynth Ecogenics (Balgach, Switzerland) to identify and test nuclear SSR loci using a next‐generation sequencing‐based enrichment protocol. Of the 285 identified microsatellite loci, 48 had primers designed and tested, which returned six polymorphic markers that were amplified consistently. For each of the six loci, PCR was carried out as follows: Each forward primer had a 5′—AGGTTTTCCCAGTCACGACGTT—3′ M13 sequence attached at the 5′ end for subsequent detection purposes. DNA was amplified in a total volume of 20 μL comprising the following reaction mixture: 1.5 μL DNA, 1X PCR buffer (Bioron, Germany), 5 μM of each primer (0.2 mM of each dNTP (VWR International)), 0.25 μM M13 oligo with a fluorescent dye attached, and 0.25 U Taq DNA polymerase (Bioron). PCR conditions for all markers except JC035 involved the following steps: Initial denaturation at 95°C for 5 min, followed by 10 cycles of denaturation at 94°C for 30 s; annealing at 55°C for 60 s; and extension at 72°C for 30 s. This was followed by 26 cycles of denaturation at 94°C for 30 s; annealing at 53°C for 60 s; and extension at 72°C for 30 s. A final extension step was carried out at 72°C for 10 min. PCR conditions for JC035 were as follows: Initial denaturation at 95°C for 5 min, followed by 32 cycles of denaturation at 94°C for 30 s; annealing at 50°C for 60 s; and extension at 72°C for 30 s. A final extension step was carried out at 72°C for 10 min. PCR products were run on a Licor 4300 DNA sequencer, and the allele sizes were scored against a size marker standard.

### 2.2 Data Cleaning

Marker data were cleaned and checked: Monomorphic loci, those with more than 10% missing data, and those out of Hardy–Weinberg Equilibrium (HWE) in more than half of all populations were removed using GenAlEx (Peakall and Smouse 2012). The SSR data were screened for null alleles using Microchecker (Van Oosterhout et al. 2004). Finally, genotype data were screened for clonality between pairs of individuals within the same population, due to known conservation efforts that have used plants grown from cuttings in populations CD and *Hemi* (R Core Team 2021; Wickham et al. 2023). One individual was removed when two individuals within a population shared an exact genotype across all loci, but when genotypes were shared between individuals in different populations, both individuals were retained for the analyses.

### 2.3 Data Analyses: Descriptive Statistics and Fixation Indexes

The following descriptive statistics were calculated for each population using GenAlEx (Peakall and Smouse 2012): Average number of individuals (*N*), number of alleles (*N*_a_), observed (*H*_o_), and expected heterozygosity (*H*_e_), the inbreeding fixation index (*F*_is_) and the standard error for the inbreeding fixation index (SE *F*_is_). Both Wright's standard fixation index (*F*_st_) and Wright's adjusted fixation index ($F_{\mathrm{st}}^{\prime }$) were calculated using GenAlEx (Peakall and Smouse 2012), both of which are reported here, following the recommendation of Peakall and Smouse (2012). Theoretically, *F*_st_ values range from 0 to 1, with 0 representing a complete lack of population differentiation, or panmixia, and 1 representing complete isolation (Wright 1984). Generally, *F*_st_ values can be interpreted with the threshold values: < 0.05 indicating little differentiation, 0.05–0.15 indicating moderate differentiation, 0.15–0.25 indicating a large degree of differentiation, and > 0.25 indicating extreme differentiation (Hartl 2020). To evaluate the genetic differentiation within regions, both fixation indices were calculated for all pairs of populations within each region. Furthermore, to compare the genetic differentiation among regions, the arithmetic means of all pairwise comparisons of populations in different regions were calculated. Finally, M‐Ratios (Garza and Williamson 2001) were calculated using the “dplyr” package (Wickham et al. 2023) for each SSR locus and population to evaluate whether there were genetic indications of recent population bottlenecks. M‐Ratios are calculated as the number of unique SSR genotypes divided by the range of allele sizes per locus, with M‐Ratios of less than 0.68 indicating the likelihood of a recent reduction in population size (Garza and Williamson 2001).

### 2.4 Data Analyses: Population Structure and Genetic Differentiation

A hierarchical analysis of molecular variance (AMOVA) was performed using GenAlEx (Peakall and Smouse 2012) with 9999 permutations and within‐individual variability suppressed. Tests for Isolation by Distance (IBD), in addition to Principal Coordinate Analyses (PCoA), were both performed using GenAlEx (Peakall and Smouse 2012). IBD was evaluated by running a Mantel test comparing log‐transformed geographic distances with pairwise, individual‐by‐individual genetic distances for all populations, as well as for only Scottish populations due to their larger geographic distribution than the other population centers. We tested for genetic structure using the STRUCTURE software v.2.3.4 (Pritchard et al. 2000). The Markov chain Monte Carlo (MCMC) method used a burn‐in length of 10,000 steps, followed by 10,000 steps for estimation. Each simulation was replicated with 20 iterations for each *K* value between 2 and 21, from which mean values were calculated. These data were uploaded to STRUCTURE Harvester (Earl and vonHoldt 2012) and StructureSelector (Li and Liu 2018) to estimate the most likely *K* value using the delta *K* method (Evanno et al. 2005) and to CLUMPAK (Kopelman et al. 2015) to summarize the *Q* matrices for all 20 iterations of each *K* into a single *Q*‐matrix. The returned *Q*‐matrices for each *K* value were processed into spatial objects using the raster package for R (Hijmans 2023) and plotted as pie charts in ArcMap. We report the results for *K* = 3, 4, and 5 for both SNP and SSR datasets.

### 2.5 Subspecies Exclusions

The population of *Hemi* was removed when calculating pairwise *F*_st_ values and performing the AMOVA, IBD, and PCoA analyses for the following two reasons. First, only 39.2% of SNP and 50% of SSR loci were polymorphic for *Hemi*. Second, *Hemi* was highly differentiated from the other populations, with pairwise *F*_st_ values between *Hemi* and other populations ranging from 0.359 to 0.481 for SNP data and from 0.238 to 0.499 for SSR data. Inclusion of *Hemi* in AMOVA, IBD, and PCoA would, therefore, have obscured finer‐scale differences between the other populations. Conversely, *Nana* was included in all calculations. Unlike *Hemi, Nana* had an acceptable percentage of polymorphic loci, and including *Nana* in pairwise *F*_st_ values, AMOVA, and PCoA resulted in only minor differences in these values and analyses.

## 3 Results

### 3.1 Data Cleaning

Fourteen SNP loci with more than 10% missing data and a further nine monomorphic loci were removed. An additional 10 SNP loci were removed for being significantly out of HWE (*p* < 0.05) in more than half of all populations, leaving a total of 41 loci for analysis. Two SSR loci were removed due to null alleles in more than half of all populations, leaving 4 SSR loci for analysis. Twelve individuals from the SNP dataset shared genotypes across all loci, sharing five genotypes among them. These were in populations CD, *Nana*, and *Hemi*, and none shared a genotype with any individuals from other populations—in each case, only one individual per genotype was retained for subsequent analyses (Table A3). Thirty‐six individuals in the SSR dataset shared 16 genotypes among them. Of these 18 pairs of individuals, 10 pairs were individuals from the same population, and in each case, one individual was removed at random. The other 8 pairs were among individuals from different populations, and both were retained. These were in populations *Hemi, Nana*, CD, PD, and LM, and again, only one representative of each genotype was retained (Table A4). Finally, one individual from WW was removed from the SNP dataset due to being an extreme outlier, as it differed in virtually every locus from all other individuals in that population, suggesting the possibility of an extraction or sequencing error. This left 277 and 304 individuals in the SNP and SSR data, respectively (Table 1).

### 3.2 Descriptive Statistics

In the SNP dataset, the percentage of polymorphic loci within populations ranged from 68.3% (*Nana*) to 97.6% (CD), with an average of 88.4%. *F*_is_ values from the SNP dataset had an overall mean of 0.025 and a range from −0.221 (*Hemi*) to 0.108 (BF) (Table 2). All populations in the SSR dataset had 100% polymorphic loci except for *Hemi*, which had 50%. *F*_is_ values from the SSR dataset had a mean of −0.002 and a range from −0.159 (*Nana*) to 0.171 (BG) (Table 3). Six populations (BG, CD, DH, FK, GA, and PD) had positive *F*_is_ values for both SNP and SSR datasets; eight populations (AR, BF, BH, LM, *Nana*, TF, TY, and WW) had one positive value and one negative value between the SNP and SSR datasets; the remaining four populations (BT, *Hemi*, IN, and MB) had negative values for both datasets. The average M‐Ratios across loci ranged from 0.191 (TF) to 0.278 (DH) (Table 3). However, the low number of loci in our study and the variable numbers of individuals per population may have been partially responsible for these low values (Garza and Williamson 2001).

**TABLE 2:** Mean descriptive statistics for SNP data across all 41 loci for each population.

Pop	*N*	*N*_a_	*H*_o_	*H*_e_	u*H*_e_	*F*_is_	*F*_is_ SE	% Polymorphic
AR	11.9	1.902	0.298	0.327	0.341	0.076	0.054	90.24%
BF	9.9	1.927	0.244	0.292	0.307	0.108	0.054	92.68%
BG	22.2	1.951	0.289	0.305	0.312	0.051	0.042	95.12%
BH	11.8	1.902	0.274	0.293	0.306	0.090	0.053	90.24%
BT	9.9	1.854	0.279	0.277	0.292	−0.001	0.054	85.37%
CD	19.8	1.976	0.299	0.315	0.323	0.022	0.046	97.56%
DH	21.9	1.902	0.265	0.281	0.288	0.030	0.043	90.24%
FK	21.8	1.951	0.267	0.288	0.295	0.074	0.045	95.12%
GA	22.9	1.902	0.275	0.310	0.317	0.084	0.050	90.24%
*Hemi*	10.9	1.439	0.201	0.160	0.168	−0.221	0.043	43.90%
IN	5.9	1.829	0.340	0.295	0.323	−0.145	0.060	82.93%
LM	22.8	1.902	0.291	0.297	0.304	0.020	0.042	90.24%
MB	6.9	1.829	0.336	0.330	0.356	−0.018	0.067	82.93%
*Nana*	8.0	1.683	0.220	0.227	0.242	0.023	0.069	68.29%
PD	21.9	1.878	0.259	0.295	0.302	0.078	0.038	87.80%
TF	21.5	1.902	0.286	0.268	0.275	−0.046	0.040	90.24%
TY	14.7	1.927	0.252	0.267	0.276	0.033	0.050	92.68%
WW	8.9	1.805	0.263	0.277	0.293	0.044	0.066	80.49%
Mean	15.2	1.859	0.274	0.284	0.296	0.025	n/a	85.91%
SE	0.23	0.013	0.007	0.006	0.007	0.012	n/a	2.94%

Abbreviations: *F*_is_, inbreeding fixation index; *F*_is_ SE, standard error of fixation index; *H*_e_, expected heterozygosity; *H*_o_, observed heterozygosity; *N*, average number of individuals with valid data per locus; *N*_a_, number of alleles; u*H*_e_, unbiased expected heterozygosity, % Polymorphic, percent of total loci which are polymorphic for each population.

**TABLE 3:** Mean descriptive statistics for SSR data across all 4 loci for each population.

Pop	*N*	*N*_a_	*H*_o_	*H*_e_	u*H*_e_	*F*_is_	*F*_is_ SE	M‐Ratio
AR	11	5.000	0.614	0.574	0.602	−0.074	0.009	0.253
BF	10	5.000	0.700	0.646	0.680	−0.099	0.166	0.266
BG	30	6.750	0.558	0.655	0.666	0.171	0.090	0.260
BH	11	4.750	0.568	0.548	0.574	−0.068	0.099	0.213
BT	8	4.250	0.563	0.557	0.594	−0.025	0.092	0.232
CD	35	7.500	0.586	0.608	0.617	0.051	0.094	0.233
DH	27	6.000	0.574	0.663	0.675	0.158	0.069	0.278
FK	26	6.500	0.558	0.610	0.622	0.077	0.068	0.212
GA	24	7.250	0.635	0.678	0.692	0.060	0.046	0.248
*Hemi*	7	2.000	0.321	0.281	0.302	−0.117	0.184	0.314
IN	6	3.250	0.500	0.510	0.557	−0.003	0.119	0.269
LM	30	5.000	0.608	0.582	0.592	−0.065	0.046	0.249
MB	6	4.500	0.750	0.688	0.750	−0.105	0.218	0.223
*Nana*	6	3.750	0.625	0.507	0.553	−0.159	0.137	0.219
PD	24	5.750	0.594	0.658	0.672	0.098	0.049	0.215
TF	25	5.250	0.560	0.582	0.594	0.007	0.081	0.191
TY	15	4.000	0.583	0.588	0.609	−0.018	0.080	0.226
WW	9	4.750	0.611	0.603	0.639	−0.032	0.096	0.214
Mean	17.2	5.069	0.584	0.585	0.610	−0.002	n/a	0.238
SE	1.1	0.260	0.029	0.026	0.027	0.024	n/a	0.029

*Note:* All loci for all populations except HEM were 100% polymorphic.

Abbreviations: *F*_is_, fixation index; *F*_is_ SE, standard error for fixation index; *H*_e_, expected heterozygosity; *H*_o_, observed heterozygosity; M‐Ratio, average of M‐Ratio values calculated for each locus; *N*, number of individuals; *N*_a_, number of alleles; u*H*_e_, unbiased expected heterozygosity.

### 3.3 Population Differentiation Within Regions

Within S. England and Scotland, *F*_st_ estimates were small but significant, indicating minor but detectable genetic differences among populations. Within the Lake District, estimates were not significant. In both SNP and SSR datasets, estimates of *F*_st_ and $F_{\mathrm{st}}^{\prime }$ among populations were lower in the Lake District and S. England than in Scotland. The average $F_{\mathrm{st}}^{\prime }$ values for SNP and SSR datasets were 0.029 and 0.069 for S. England, 0.025 and 0.035 for the Lake District, and 0.072 and 0.165 for Scotland, respectively. Pairwise estimates of $F_{\mathrm{st}}^{\prime }$ for the SNP and SSR datasets within each region ranged from 0.028 (BH‐PD and DH‐PD) to 0.031 (DH‐BH) for S. England; 0.011 (BT‐TF) to 0.022 (BT‐BF) for the Lake District; and 0 (MB‐GA) to 0.134 (*Nana‐*WW) for Scotland (Tables 4 and 5). All $F_{\mathrm{st}}^{\prime }$ values were slightly higher than *F*_st_ values due to being corrected by the maximum possible differentiation within populations; $F_{\mathrm{st}}^{\prime }$ values are more suited for comparing different marker types for this reason.

**TABLE 4:** Pairwise *F*_st_ (bottom) and $F_{\mathrm{st}}^{\prime }$ values among all populations calculated from the SNP dataset.

LD	BF		0.031	0.027	0.089	0.113	0.185	0.142	0.063	0.113	0.124	0.110	0.161	0.117	0.066	0.145	0.095	0.164
LD	BT	0.022		0.016	0.086	0.094	0.151	0.094	0.030	0.081	0.066	0.071	0.134	0.075	0.033	0.104	0.078	0.086
LD	TF	0.019	0.011		0.097	0.095	0.143	0.110	0.055	0.081	0.074	0.076	0.089	0.077	0.083	0.110	0.075	0.087
SE	BH	0.061**	0.06**	0.067***		0.044	0.040	0.175	0.068	0.100	0.134	0.127	0.192	0.164	0.110	0.192	0.132	0.156
SE	DH	0.079***	0.066**	0.067***	0.031*		0.040	0.181	0.080	0.094	0.136	0.080	0.163	0.113	0.100	0.157	0.102	0.118
SE	PD	0.129***	0.105***	0.1***	0.028*	0.028*		0.195	0.113	0.110	0.145	0.117	0.184	0.157	0.127	0.214	0.145	0.152
SC	AR	0.095***	0.064**	0.075***	0.117***	0.124***	0.133***		0.074	0.074	0.063	0.084	0.096	0.082	0.054	0.104	0.144	0.153
SC	BG	0.042**	0.02	0.037**	0.045**	0.054***	0.076***	0.048**		0.021	0.040	0.011	0.086	0.044	0.007	0.064	0.051	0.072
SC	CD	0.076***	0.055**	0.056***	0.067***	0.065***	0.075***	0.049**	0.014*		0.055	0.018	0.057	0.080	0.043	0.091	0.077	0.038
SC	FK	0.086***	0.046**	0.052***	0.092***	0.095***	0.101***	0.043**	0.027**	0.037**		0.028	0.060	0.074	0.009	0.081	0.118	0.123
SC	GA	0.075***	0.049**	0.053***	0.086***	0.055***	0.08***	0.056**	0.007	0.012	0.019*		0.051	0.026	−0.009	0.049	0.074	0.062
SC	IN	0.11***	0.093*	0.062*	0.131***	0.114***	0.127***	0.064*	0.056*	0.038*	0.041*	0.035*		0.124	0.054	0.109	0.121	0.093
SC	LM	0.081***	0.052**	0.054***	0.112***	0.079***	0.108***	0.055**	0.029*	0.054**	0.051**	0.018*	0.085**		0.030	0.096	0.065	0.092
SC	MB	0.044*	0.023	0.057*	0.073**	0.069**	0.086***	0.035*	0.005	0.028	0.006	0	0.036	0.02		0.071	0.079	0.101
SC	*Nana*	0.104***	0.076*	0.079**	0.137***	0.113***	0.153***	0.073**	0.043*	0.064**	0.058**	0.034*	0.079*	0.068**	0.05*		0.133	0.185
SC	TY	0.066***	0.055**	0.053**	0.091***	0.072***	0.101***	0.098***	0.034**	0.053**	0.082***	0.051**	0.084**	0.045**	0.054*	0.096***		0.080
SC	WW	0.114***	0.06*	0.061**	0.107***	0.083***	0.105***	0.103***	0.048**	0.026*	0.085***	0.042*	0.064*	0.064**	0.068*	0.134***	0.056**	
	Pop	BF	BT	TF	BH	DH	PD	AR	BG	CD	FK	GA	IN	LM	MB	*Nana*	TY	WW
Region		LD	LD	LD	SE	SE	SE	SC	SC	SC	SC	SC	SC	SC	SC	SC	SC	SC

*Note:* See Table 1 for population abbreviations, and the region that each population is included in is also included, with “LD” being Lake District, “SE” being S. England, and “SC” being Scotland. *p*‐values for $F_{\mathrm{st}}^{\prime }$ values are represented by asterisks, with * indicating a *p*‐value between 0.05 and 0.005, ** indicating a *p*‐value between 0.0049 and 0.0005, and *** indicating a *p*‐value smaller than 0.0005.

**TABLE 5:** Pairwise *F*_st_ (bottom) and $F_{\mathrm{st}}^{\prime }$ values among all populations calculated from the SSR dataset.

LD	BF		0.059	−0.001	0.310	0.256	0.218	0.416	0.245	0.288	0.260	0.201	0.473	0.263	0.018	0.432	0.373	0.136
LD	BT	0.021		0.012	0.157	0.157	0.114	0.238	0.149	0.082	0.215	0.099	0.281	0.114	−0.028	0.334	0.174	−0.012
LD	TF	0.000	0.005		0.268	0.215	0.195	0.385	0.227	0.265	0.272	0.232	0.372	0.209	0.107	0.436	0.299	0.070
SE	BH	0.116***	0.066*	0.111***		0.144	0.018	0.110	0.087	0.056	0.039	0.105	0.286	0.125	0.216	0.143	0.218	0.145
SE	DH	0.083***	0.056**	0.079***	0.053**		0.044	0.185	0.254	0.217	0.249	0.273	0.269	0.228	0.135	0.370	0.179	0.138
SE	PD	0.071***	0.041*	0.071***	0.006	0.015*		0.122	0.107	0.080	0.127	0.110	0.234	0.065	0.105	0.204	0.089	0.111
SC	AR	0.15***	0.096**	0.155***	0.046*	0.066***	0.044**		0.198	0.110	0.183	0.178	0.257	0.277	0.177	0.259	0.245	0.284
SC	BG	0.08***	0.054**	0.084***	0.032*	0.084***	0.035**	0.071***		0.049	0.086	0.034	0.290	0.098	0.141	0.072	0.190	0.087
SC	CD	0.103***	0.032*	0.104***	0.023*	0.077***	0.029**	0.043**	0.018*		0.094	0.025	0.196	0.108	0.086	0.077	0.117	0.122
SC	FK	0.092***	0.084***	0.107***	0.015	0.087***	0.045***	0.071***	0.031**	0.036***		0.070	0.233	0.218	0.166	0.081	0.270	0.195
SC	GA	0.063***	0.034*	0.083***	0.038*	0.087***	0.035***	0.062***	0.011	0.009	0.024*		0.211	0.137	0.037	0.097	0.162	0.107
SC	IN	0.177***	0.119**	0.156***	0.124**	0.099***	0.086**	0.107***	0.108***	0.079**	0.093**	0.076**		0.327	0.243	0.241	0.095	0.267
SC	LM	0.098***	0.046*	0.085***	0.052**	0.084***	0.024*	0.112***	0.037**	0.043***	0.086***	0.049***	0.137***		0.233	0.221	0.121	0.141
SC	MB	0.005	0.000	0.037	0.076**	0.04*	0.031	0.059*	0.043*	0.029	0.055*	0.010	0.084*	0.082**		0.281	0.167	0.002
SC	*Nana*	0.162***	0.142***	0.183***	0.062*	0.136***	0.076**	0.109**	0.027	0.031	0.033	0.035*	0.107*	0.093**	0.098*		0.237	0.277
SC	TY	0.134***	0.069*	0.119***	0.089***	0.063***	0.032*	0.097***	0.068***	0.045**	0.104***	0.056***	0.039	0.048**	0.056*	0.098**		0.178
SC	WW	0.046*	0.000	0.027	0.057*	0.047**	0.038*	0.108***	0.03*	0.046*	0.073***	0.035*	0.106**	0.055*	0.001	0.111**	0.067**	
	Pop	BF	BT	TF	BH	DH	PD	AR	BG	CD	FK	GA	IN	LM	MB	*Nana*	TY	WW
Region		LD	LD	LD	SE	SE	SE	SC	SC	SC	SC	SC	SC	SC	SC	SC	SC	SC

*Note:* See Table 1 for population abbreviations, and the region that each population is included in is also included, with “LD” being the Lake District, “SE” being S. England, and “SC” being Scotland. *p*‐values for $F_{\mathrm{st}}^{\prime }$ values are represented by asterisks, with * indicating a *p*‐value between 0.05 and 0.005, ** indicating a *p*‐value between 0.0049 and 0.0005, and *** indicating a *p*‐value smaller than 0.0005.

### 3.4 Differentiation Among Regions

Regions showed markedly different patterns of fixation index values, with comparisons between populations in Scotland and S. England having the highest $F_{\mathrm{st}}^{\prime }$ values in the SNP dataset ($F_{\mathrm{st}}^{\prime }$ = 0.137) and those between Scotland and the Lake District having the highest values in the SSR dataset ($F_{\mathrm{st}}^{\prime }$ = 0.231). The average of all pairwise $F_{\mathrm{st}}^{\prime }$ values comparing regions for SNP data ranged from 0.025 to 0.137 ($F_{\mathrm{st}}^{\prime }$, Lake District‐Lake District and Scotland‐S. England, respectively) (Table 6). For SSR data, these comparisons ranged from 0.023 to 0.231 ($F_{\mathrm{st}}^{\prime }$, Lake District‐Lake District and Scotland‐Lake District, respectively) (Table 7).

**TABLE 6:** Average of pairwise *F*_st_ (top) and adjusted $F_{\mathrm{st}}^{\prime }$ (bottom) values among all populations within and between the three regions for SNP data.

*F*_st_	Scotland	Lake district	S. England
Scotland	0.050		
Lake District	0.064	0.017	
S. England	0.095	0.081	0.029

$F_{\mathrm{st}}^{\prime }$	Scotland	Lake district	S. England
Scotland	0.072		
Lake District	0.093	0.025	
S. England	0.137	0.117	0.041

*Note:* Values were calculated as the means of all pairwise comparisons either within a region or between different regions.

**TABLE 7:** Average of pairwise *F*_st_ (top) and adjusted $F_{\mathrm{st}}^{\prime }$ (bottom) values among all populations within and between the three regions for SSR data.

*F*_st_	Scotland	Lake district	S. England
Scotland	0.067		
Lake District	0.136	0.045	
S. England	0.079	0.044	0.027

$F_{\mathrm{st}}^{\prime }$	Scotland	Lake district	S. England
Scotland	0.165		
Lake District	0.231	0.023	
S. England	0.163	0.210	0.069

*Note:* Values were calculated as the means of all pairwise comparisons either within a region or between different regions.

### 3.5 Population Structure: AMOVA

The hierarchical AMOVA's for both SNP and SSR data ascribed the majority of the observed genetic variance to that within a population (92% for both SNP and SSR datasets). The variance among populations was significant for both SNP (*F*_st_ = 0.079; *p* < 0.001) and SSR data (*F*_st_ = 0.076; *p* < 0.001) but accounted for only 4% of the observed variation in both datasets. Similarly, the variance among regions was also significant for both SNP (*F*_rt_ = 0.043; *p* < 0.001) and SSR data (*F*_rt_ = 0.037; *p* < 0.001) and accounted for 4% of the observed variation in both datasets.

### 3.6 Isolation by Distance

There were significant, positive correlations between log‐transformed geographic and genetic distances over all populations in both SNP (*R* ^2^ = 0.0042, *p* = 0.001) and SSR (*R* ^2^ = 0.0079, *p* = 0.001) datasets. Within the Scottish region, both SNP (*R* ^2^ = 0.007, *p* = 0.001) and SSR (*R* ^2^ = 0.0029, *p* = 0.002) datasets detected significant IBD. Despite having significant *p*‐values, these correlations were very weak.

### 3.7 Population Structure: PCoA

**FIGURE 2:**
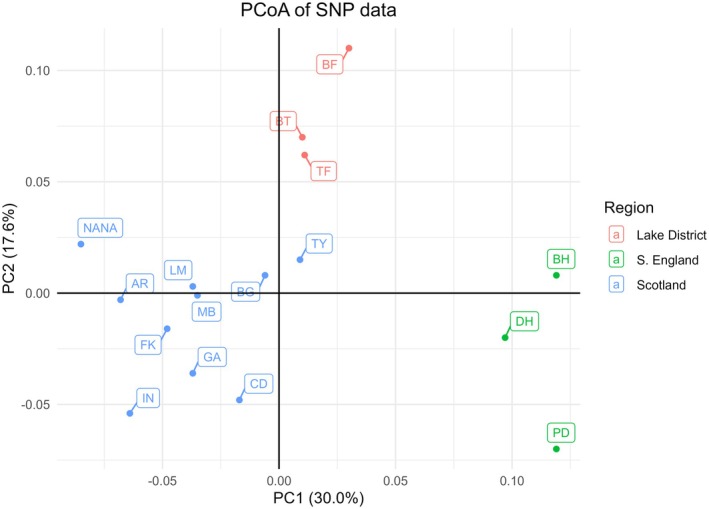
Principal coordinate analysis (PCoA) of SNP dataset. Population abbreviations are given in Table 1 and regions are color‐coded.

**FIGURE 3:**
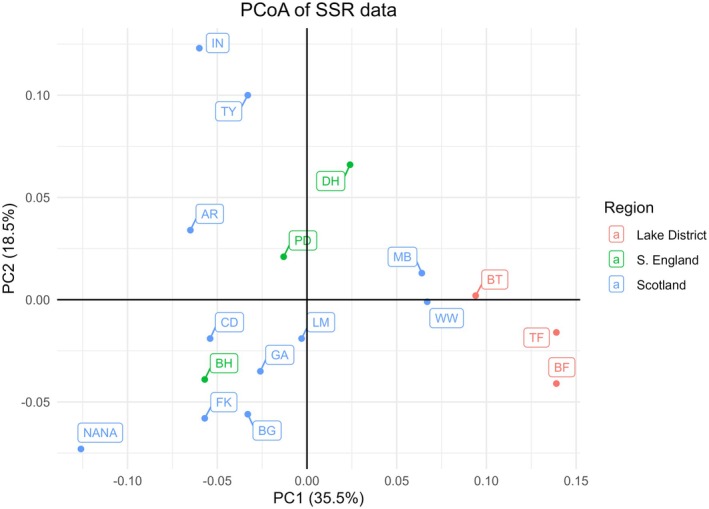
Principal coordinate analysis (PCoA) of the SSR dataset. Population abbreviations are given in Table 1 and regions are color‐coded.

The first three principal coordinates accounted for 30.0%, 17.6%, and 13.1% of the observed variation in the SNP and 35.5%, 18.5%, and 14.1% of the observed variation in the SSR datasets. In the SNP dataset (Figure 2), populations from different regions clustered closely together, while in the SSR dataset (Figure 3), the regional grouping was less pronounced, with Lake District populations grouping together, but Scottish and S. English populations overlapping.

### 3.8 Population Structure: STRUCTURE

**FIGURE 4:**
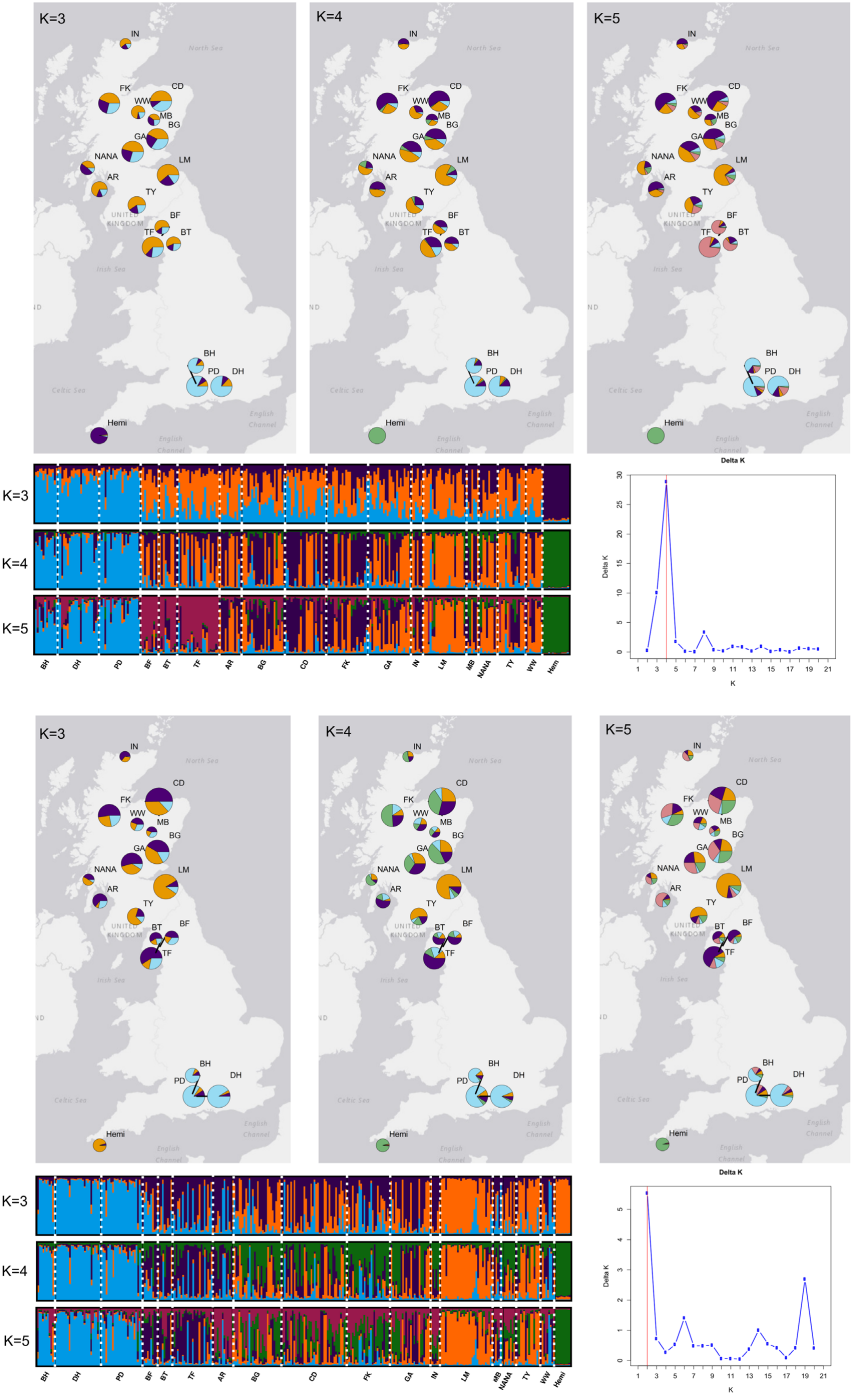
Results from STRUCTURE runs based on SNP (top) and SSR (bottom) datasets for *K* = 3, *K* = 4, and *K* = 5. Pie charts display locations of sampled populations and proportional membership of each population to each genetic group, with pie chart colors corresponding to the colors from the STRUCTURE plots shown in the bottom left of each panel. The size of each chart is proportional to the number of sampled individuals from that population. Bottom left: Bar graphs display proportional membership of each individual to each genetic group, where each bar is an individual, and white dotted lines demark the different populations that are shown along the *x*‐axis, population abbreviations given in Table 1. Bottom right: STRUCTURE Delta *K* graphs, showing the change in log‐likelihood (L(*K*)) values between runs with different values of *K*.

In the STRUCTURE analyses, we found that the optimal *K* value for the SNP dataset was *K* = 4, and for the SSR dataset was *K* = 2, with *K* = 6 as the second most likely *K* value (Figure 4). In both datasets, the S. England populations grouped strongly together, and *Hemi* is ascribed almost entirely to a single genetic group. Lake District populations were grouped together and mostly excluded populations from the other regions at *K* = 5 (SNP) and *K* = 4 (SSR). Scottish populations showed some sign of sub‐structuring into two groups, particularly in the SSR dataset, with those populations in the Highlands (IN, FK, CD, WW, MB, GA, AR) and those in the Borders (TY, LM) grouping together, although both genetic groups were present throughout. This pattern was notable at SNP *K* = 5 and at SSR *K* = 3–5. Mean Ln(P) values for *K* = 3, *K* = 4, and *K* = 5 were −11,485.4, −11,225.7, and −11,178.5 for the SNP dataset and −6074.4, −6022.7, and −5973.7 for the SSR data.

## 4 Discussion

Our analyses of two types of neutral genetic markers in UK juniper found significant genetic structure among UK populations. First, we found a clear genetic distinctiveness of *Hemi* from all other populations of *J. communis* and *Nana*, which supports previous subspecies classifications of *J. communis* spp. *hemisphaerica* (Thomas et al. 2007). *Nana*, while distinct, was more closely related to *J. communis*. Second, we found significant among‐population and among‐region structuring, with regional groupings showing different patterns of genetic diversity and variable levels of among‐population differentiation. M‐Ratios calculated from the SSR dataset also suggest that all sampled populations have been experiencing bottlenecks, which is likely the outcome of more than a century of fragmentation and decline (Sullivan 2003; Thomas et al. 2007).

All analyses suggested significant genetic structure was present among populations and among regions, with the exception of the populations in the Lake District, which were not significantly differentiated within the region. This structure is evident in the PCoA results from the SNP dataset, which shows notable groupings by region (Figure 2). The SNP dataset indicated clearer distinctions than the SSR dataset, likely due to the fact that SSR markers have higher per‐locus mutation rates, which occur as step‐wise changes that result in greater heterozygosity and less clear genetic distinctions among populations. STRUCTURE found an optimal number of genetic groups (*K* value) of four and two for the SNP and SSR datasets, respectively. At *K* = 4 in the SNP dataset, both *Hemi* and S. English populations were distinct, while the Lake District and Scottish populations were split between the remaining two groups, with populations in the Scottish Borders being more similar to those in the Lake District than either was to the more northern stands. Although structure was evident, the slight to moderate *F*_st_ and $F_{\mathrm{st}}^{\prime }$ estimates suggest that some gene flow still occurs among regions. It is possible that these estimates are reflective of a historically larger metapopulation that has since declined and become fragmented. If that is the case, we can expect genetic differentiation to increase with time. Studies of other European tree species have generally found low levels of genetic differentiation, with *F*_st_ rarely exceeding 0.10; higher values were interpreted as very large interpopulation differentiation (Dobeš et al. 2017; Hamrick et al. 1992; Müller‐Starck et al. 1992). Our estimates, therefore, represent a notable degree of genetic differentiation.

The three UK regions had different patterns of within‐region genetic structure (Tables 4, 5, 6, 7). Lake District populations showed no significant genetic structure, suggesting gene flow among populations sufficient to maintain outcrossing. Southern English populations were very similar to one another (Tables 6 and 7), but two of the three sites were significantly differentiated, indicating that the degree of genetic isolation was more advanced. Scottish populations were generally more differentiated from one another than populations within either of the other regions were from each other (Tables 6 and 7). To some extent, this is to be expected, given the greater number of sampled populations and larger inter‐population distances in Scotland compared to the other regions. Nonetheless, the higher diversity and differentiation of Scottish populations are indicative of a fragmented landscape with substantial barriers to gene flow between populations (Oostermeijer and De Knegt 2004; Provan et al. 2008; Reim et al. 2016). A weak but significant finding of IBD within Scotland also suggests spatially constrained pollen and seed (and consequently gene) dispersal capability (Meirmans 2012). Previous work on juniper pollen and seed dispersal generally finds that local deposition (within tens of metres) is most common (Adams and Thornburg 2010; García 2001; Surso 2018); however, Hall (1990) reported the long‐range dispersal of juniper pollen as far as 180 km. The weak IBD would be consistent with predominantly local dispersal, with occasional long‐distance events.

There are some caveats to our results that are worth mentioning. The small number of loci, particularly in the SSR dataset, and the uneven sample numbers across different populations may have caused underestimates of both allelic richness and diversity indices and their standard errors (Carling and Brumfield 2007; Garza and Williamson 2001; Kalinowski 2002; Rosenberger et al. 2021). Furthermore, since the SNP and SSR markers differ substantially in their mutation processes and allelic diversity, and since they were filtered differently (based on missing data and HWE for the former, and null alleles for the latter), F_is_ values should be treated with a degree of caution and only be compared among populations within marker datasets. Although the SSR dataset ultimately had only four loci, this was balanced to some extent by its higher allelic richness per locus, which can improve the precision of diversity estimates (Kalinowski 2002). Similarly, although 41 SNP loci are small compared to studies of model organisms, Santure et al. (2010) demonstrated that 20 microsatellites had about the same resolution of information as 50 SNPs and that combining these datasets could improve the overall resolution. With these limitations in mind, and provided our results are interpreted with a degree of caution, we are confident that the broad‐scale differences observed here are biologically sound.

Previous work by García et al. (2018) comparing SNP and SSR markers in *J. phoenicea* found that the former were more informative regarding demographic inferences, whereas the latter is more suited to parentage and assignment studies. They noted that the larger per‐locus information content of SSR markers yields higher diversity estimates, but either a larger number of genome‐wide SNPs or a smaller number of SSRs (147 and 19, respectively, in their case) performed adequately at estimating genetic diversity. We also found higher diversity estimates using SSR markers than SNPs (mean H_o_ over all populations = 0.584 and 0.274, respectively). Besides these expected differences, our results were largely similar for both types of markers in terms of the patterns of differentiation. For example, both SNP and SSR datasets found no significant *F*_st_ values among Lake District populations but multiple significant values among Southern English and Scottish populations, a very low average *F*_is_ value across loci and populations, nearly identical AMOVA results, and similar genetic groupings using STRUCTURE.

Our results suggest that over a century of habitat fragmentation has had a detectable negative impact, impeding gene flow among UK juniper stands and resulting in genetic isolation among many of the sampled populations. Although the longevity of juniper trees allows the possibility that some of the sampled individuals may have been progeny of members of a historically larger, more interconnected metapopulation, the decline of population sizes and extinction of populations in the United Kingodm has continued. Our results suggest that we may be approaching the end of the “lag‐time” between fragmentation occurring and the resulting genetic impacts, with several populations now showing signs of genetic isolation. In this case, our results would be an underestimate of the effects of the current level of fragmentation, and we should expect juniper genetic diversity to decline further without remedial action.

Our findings are consistent with previous studies of juniper population genetics in the British Isles in that they have all found evidence of significant genetic differentiation and limited gene flow between remnant stands (Merwe et al. 2000; Provan et al. 2008; Reynolds 2022). Although direct comparisons between diversity indices from population genetic studies that use different marker types and sampling regimes are not possible, there is a shared pattern in the population genetics of European *J. communis*. By contrast to those in the British Isles, studies on mainland Europe have generally found low levels of genetic differentiation with an absence of a geographic pattern to the observed genetic diversity (Hantemirova et al. 2012, 2017; Knyazeva and Hantemirova 2020; Oostermeijer and De Knegt 2004; Reim et al. 2016; Vanden‐Broeck et al. 2011). Two notable exceptions to this pattern are the significant differentiation of populations in Russia's far east and Caucasus from those in Western Europe (Hantemirova et al. 2012, 2017; Knyazeva and Hantemirova 2020), and the high degree of differentiation, but lack of geographic signals, that Michalczyk et al. (2010) reported between populations from across central Europe. These studies may be highlighting the geographic distances that juniper gene flow can span in favorable conditions: The latter studies, which found some genetic differentiation, observed it over larger areas, whereas those that didn't find patterns of differentiation were generally conducted across smaller areas.

Juniper is a pioneer species that can thrive in tundra ecosystems (Hantemirova et al. 2017; Michalczyk et al. 2010; Unterholzner et al. 2020), and it is, therefore, often studied to discern the biogeography of hardy plant species after the Last Glacial Maximum (LGM) (Hantemirova et al. 2017; Michalczyk et al. 2010). The observed pattern of continental European juniper populations having very little genetic differentiation and a lack of geographic signals is hypothesized to be due to repeated fragmentation and expansion events from glacial refugia across Europe, allowing for gene flow between or even during glacial events that homogenized these populations (Hantemirova et al. 2012, 2017; Knyazeva and Hantemirova 2020; Michalczyk et al. 2008, 2010). By contrast, the high differentiation that studies from the British Isles report is most often interpreted only as evidence that barriers to gene flow exist between populations (Merwe et al. 2000; Provan et al. 2008; Reynolds 2022), and only Merwe et al. (2000) have posited a hypothesis on how the area was recolonized by junipers after the LGM. This hypothesis was based on their findings of three genetic groups in Great Britain: One in Cornwall and Southern Wales, a second in Southern England and Northern Wales, and a third in Eastern and Northern England. They posited different migration events from Spain, France, and from across Doggerland (now the North Sea), respectively. The findings of Reynolds (2022) did not support this hypothesis, however, finding high haplotype diversity and multiple genetic groups among the junipers of Southern Wales. Although shedding light on the question of juniper's migration to Great Britain after the LGM is beyond the scope of the present study, an ongoing project will evaluate this by including a much wider range of populations from the British Isles and Eurasia.

Understanding the current genetic diversity of a species is the first step toward implementing dynamic conservation. This study has described the current genetic status of juniper stands in Britain. Our findings suggest that *J. communis* populations in Britain consist of multiple distinct genetic groups. Furthermore, populations are showing the effects of long‐term fragmentation, with significant differentiation and evidence of genetic isolation.

### 4.1 Conservation Recommendations

Juniper trees and juniper scrub habitat are “almost a habitat in [their] own right” (Wilkins and Duckworth 2011), hosting dozens of species of lichens, bryophytes, plants and animals among their branches, many of which are specialists and depend on juniper entirely (Binder and Ellis 2008; Ellis and Coppins 2009). Juniper scrub is also an important component of forest successional transitions, aiding tree recruitment and being associated with other bare‐ground plants (Wilkins and Duckworth 2011). Although juniper populations are expanding in some parts of their global range (Garza and Williamson 2001), they have been in precipitous decline in the United Kingdom for at least the past century and possibly longer (Sullivan 2003; Thomas et al. 2007; Wilkins and Duckworth 2011). This decline has been caused primarily by land‐use change, inappropriate grazing levels, and the introduction of the novel pathogen *Phytophthora austrocedri*. The last, which causes very high mortality by forming necrotic lesions in the cambium of the roots and lower branches, effectively girdling juniper trees, is possibly now the primary threat to the species, although there is some evidence of natural resistance (Green et al. 2015). However, for this adaptive potential to be realized requires gene dispersal and population regeneration are required, which our population genetic data indicate is restricted. The most effective measures to conserve juniper stands would be to ensure that they have healthy population sizes with active regeneration, so that populations can express whatever adaptive potential is present. Protecting natural stands from overgrazing and creating suitable micro‐habitats for their seedlings to establish are likely to be the most appropriate ways to help this process (De Frenne et al. 2020; Verheyen et al. 2005).

Planting new material within 2 km of existing juniper sites is strongly discouraged because of the associated biosecurity risks (Donald et al. 2021). Instead, the establishment of “satellite” populations—small, planted sites interspersed among remnant fragments, but further than 2 km away from any extant stand—may help to overcome these barriers to gene flow and to reconnect populations, thereby facilitating the adaptive potential and resilience of those populations. Such satellite sites should be composed of individuals raised on‐site, under strict biosecurity protocols, from locally sourced seeds. Future research should focus on the dynamics of pollen and seed dispersal to enable best practice in satellite plantings to reconnect population fragments. Incorporating genetics and conservation data can provide insights into the health of juniper populations, and a focus on conserving not only the trees but also the genes within them can benefit the adaptability and resilience of populations.

## Author Contributions

**J. Baker:** data curation (lead), formal analysis (lead), visualization (lead), writing – original draft (lead), writing – review and editing (equal). **J. Cottrell:** conceptualization (equal), project administration (equal), supervision (equal), writing – review and editing (equal). **R. Ennos:** conceptualization (equal), project administration (equal), supervision (equal), writing – review and editing (equal). **A. Perry:** project administration (equal), writing – review and editing (equal). **S. A'Hara:** methodology (lead). **S. Green:** methodology (equal), writing – review and editing (equal). **S. Cavers:** conceptualization (lead), project administration (lead), supervision (lead), writing – review and editing (equal).

## Conflicts of Interest

The authors declare no conflicts of interest.

## Data Availability

The genotypic data used in this paper are archived at the Environmental Information Data Centre and can be found here: (https://doi.org/10.5285/27e7df36‐d689‐4803‐93b4‐c15935693b83).

## Appendix A

**TABLE A1:** The 74 post‐filtering loci returned from the two multiplex Sequenom SNP chips from the CNRGV, including loci names, the two SNP base pair genotypes, which multiplex chip the loci were tested in, and the full sequence, with the SNP site and genotypes delineated with square brackets.

Loci	SNP genotypes	Multiplex 1 or 2	Sequence
1082_62	A/G	1	CTGCAGGTTTAGGACCCCTTTTGCCGCCCTTTGGAGGAGCCTTCTCCTTGGGCTTCTTCTC[A/G]GCAGGGCTTTTCTCTCCAACCGTCTTCTTCTCTTCAACCTTCTCCGCAGGTTTCTTCTCTGCGGGCTTCTTCTCTGCCTTGGGCGCCATTTTTCTGATCAATAGCAATCAATAAGGAAAATAAAAACGAAATCCTTAT
10210_101	T/A	2	CTGCAGCATTATCAGAGTCTGGGGTATTTCAGATTCTATGGATGTGTATTGCAGCAGGGGCAGTGAGTTATGTGGTGGCATTCCAGAGCAGATGCGCCCT[T/A]CCTGGACCTTGATCAATCTCATGGCCAGAGAAATATTGACAACTGTGGCACAGCCACCCCACTATGCATAGGGGTTATCCACAGTGGCTAGAGCATATA
11457_34	A/T	2	CTGCAGACCATCATGTTTCAGAATTTGCTTCAT[A/T]TACCAGTAAGACATCCTCGCAGATTTATTTCTTTCAGTTTTCCTTCACTCTGATGAAATAGCACTTTGATTGCATTTGGAGAATATAAAGGAATTTATCAACTTTGTTTTACCTTAATGAAATTTCATGATGCCGGCAGATTAGTGCTGGAAGCCCATTCAGATAC
11742_169	T/G	1	CTGCAGCAAAACTTCTCATCTTGCCGATCATCCTGTACGCCGACAGCGTCTTTGTCTGCTGCGTGAAACACAATGCATACGCTGGACCACCATTCACGTTCATTACTGTTTCAAACACCACAAGATGCACCAAGATCTGATTATAATTATCTAAGAATAATGCCATAA[T/G]CACAGTGGCCAAGTTCATCAGAATCATGAAA
1198_43	C/T	2	CTGCAGCTGAATAGTCTTCCTCTAATATCATTTCTTCATCTT[C/T]GAAGAAATTAAATTCAAGAACAATCTGAATAGGTTCATTCTCTTTAATGAAGTTATGCTTACCAACTTAGCCTCGTGGCTGCAAGCAGCATTCCCATGTTCATTTCTATAGCCTGCATGACTTTGCATGTAACCTCTCATCCTCATGTTGGTTCTTG
11995_176	G/A	2	TGCAGAAAATATCAATCTTCCTCCGGCACTGCTTTCCCGTTTTGACTTAATGTGGCTGATACTTGACAAAGCTGACATGGATAGCGACTTGGAAATGGCTAGACACGTCTTATATGTGCACCAAAAGTGCGAGCCACCTCCTTTGGGTTTTGCTCCTCTTGATTCATCCATACT[G/A]AGGTGAGTAATCGACTGATCCTTA
1212_81	C/T	1	CTGCAGAGAAAATCAGTATTGCTCTGCATAATCCTCCAACATACACATGTAGTTGAGAAGCATTCTCTCATAACCTCCCT[C/T]CATCATTGCATCTTCATCTCACCTGGGGAGAACAGCATTATCTTTTTTGCATCTCAATCACTCACATGCACAACACTACACATTTGGCTCATCCTACTCTTGCATACACAATCAGTCTT
12138_151	C/T	1	CTGCAGATTTGGTACTATCAGATCCTTGTTTTTCTTGCTGGCACATTGAATGATCCAGAAGTTGATATAGATGCCCTTTCAATAAGGTAGTTAATCCTGATGCTTCAGGCCATTGCTTAAGAAAGGTGATTAAATATGGATATATTTATA[C/T]TTGTCTAATTTACATTTTGTTGATAATTTCAGCTATACAGAACAGTTGA
12196_162	C/T	2	CTGCAGAATTGACAATGATCTTAATCTGAAATTAAAGGAAGTAAATGAATCCTACCTTAAATCAAAATAATTTCCTCGAAGACAAAGTATAAGTCCAATGACACCAATGATCAAAAAGGGGAAGTTTGATATCACATTCAGAGTATTGGGCACGCCTATAA[C/T]ACAAACACAAATACTTGTCAATATATCTCCACAAAATG
12684_84	G/T	2	CTGCAGCTTGGCACCACATAGTCAAATACATTGGTTGACTTGAGTAGTATCTCAATCAGCTCAATGACATTCAAATTGACTCA[G/T]CACTCTAGTTGCACATTGCCTTGTACACACACCTTCCAACGCAAATACTAATGATTGGAAGACCAATGGTATTTTAATTTATATTCTATCACTTATTGACAATGTGTAATTTTTTT
13131_128	T/C	1	CTGCAGCATTTGTGATAAAAGGGGGTTTATTGGCTACTGTAAAGGTCACTGGTACACACACACTACATATGCCTATTTATGAATGTATGTAGATGATTTTGTACTTATTTTGATACCGTGCTACTTA[T/C]TGTAATCAGCTTTATGAAATTTTTCAATGTTGATATTCATGTTGCACTTTTTTTATGTTCAAGTATTTGGTA
13205_45	C/T	1	CTGCAGTGGGGAAAAGAAAAGTCACGCCTGAACAAACAAGTCGG[C/T]GAAGGAGTTTGAGCCTGAGGTCGCTAAAGAAAACATAGCCCGTGGCTCACATAAGGTGCTTGTAGACAACACAAAGTCCTACAGACGATATAAGAAGCGGAAAGGGGAAACAAATTTCCTGTGGACATTGCAAGATGAGACGGGCTGTCTAACCT
13997_141	A/G	1	CTGCAGATTTGGATCGTAATCTTTTTATAGTCTTCGAATATCTTTCTAATTGTGATGGTATAGCAATTTAGCCTTCGACTTGGTCATATATGTGTGCTAATACTTCATCGCTCCACAGTATTTATTGAATAATTTAATAT[A/G]GTCTTCAACTCATGCTCTGTGTTGTTTAGATGGCTCTGTGTTGTTAATAAATTGGTACT
14296_68	T/G	1	CTGCAGTCATCAGGGTCCCATATTTTCATTGAAAACAAAGGACAACACATAATATATGGCAACACAA[T/G]AGTATTTCATAGTAAAAAAATTACCAAATTGGTTTGGAATGTAAATTATTTTTCAGGTTTATCAGTAATACATCGTCAATTCCTCTATGGCATAAGCAACCATTTGCCTAAACCATGAAAAGAAGAGAAGAA
15494_81	C/T	2	CTGCAGAGAAAATCAGTATTGCTCTGCATAATCCTCCAACATACACATGTAGTTGAGAAGCATTCTCTCATAACCTCCCT[C/T]CATCATTGCATCTTCATCTCACCTGGGGAGAACAGCATTATCTTTTTGCATCTCAATCACTCACATGCACAACACTACACATTTGGCTCATCCTACTCTTGCATACACAATCAGTCTTT
1641_85	C/T	1	CTGCAGGTTTCTGTGAAATCTAATTGGATTTTTTCCGCAGTTTGTTCTATTGACAAATCAATTTTTGGTTCAGATTTGGTATTA[C/T]TTCAGTCTGTTCATGTTGTATTTTAAAATCTTCATCTTTCCAGATAGCTTCTAAATAGGCCAATTCTTCTTCTTGATGAGGTAATGTAGGATTGTCCTAATTTTCAATTTCATTT
16700_165	T/C	2	CTGCAGATCTCTCAACTGATTCAGAGAATAATATCTGCAATGTGAACTTTTTATTTAACTAATGTTTTGGTTACTTACCATGCAGGAAGTTTTCCTTACAGTTTGGCAGGTAGCTATTATGTATTATCCGGCTGCTTATTTTAATGAATATCTGATTGCTACTT[T/C]TCTTTTCTTACTTCTCTATGTCTGCTGAGTGAGGC
16751_50	A/G	2	CTGCAGACCAACAGTTGTAATATAATGGTACTGGGGATGTAATATATTT[A/G]GGATTCCTAGGAGTGTAACACAGCTTCATCATCACATTGCAATAAGGTGGCTCCTATTCCCAGTATGTAGCAAGAATTTGTACAACTAGCACAAAAATGTGGATATATCAAAACCAAAAGAGAGACAATTATAGAAGAAAACCCTAAAAT
16960_53	A/C	1	CTGCAGGCAGCTATTGAAAACAAAGTCACTGAAGTCTGACGCTCTGACAATG[A/C]ATTTATTTCATAGTTATCTTATCCTTTCTGTCAAAAGTATTTGGTTTTAAAAATATTTAATGGATAGGCATCTTGGCCAGACCAAAGAACAATCGATCAGGTAACCGGTTCAGACTTTATCATCCAGTGGCTGTAGTGCTGTTGAAC
17261_133	A/C	2	CTGCAGAGTTTGTGAGAAGATATTTATTATCTGATCGAAGTTTCTGTTAGCTTTCATCTGAAAATGTCAGTCCATGAATTTGTAGATTGGGATACAGATGACTTTGATGGGGTAACCGAATTCACCTGGTGC[A/C]CAAGTACTGATTTCATCTGTGTTGTGGGCTATATTTATATAACCTAATAAGTAACTTTTAGTAGCAA
17598_91	A/C	2	CTGCAGGTCACGATCTTGGAACCTATCAGGAACCAAAAAATTCTTTGAATCATTTTTTAAAGTCTGATATTTAAAATAAAGACTCGCAGA[A/C]GCATGAGTCCTCATAAATAACATTCGTAGTACCCAAATCACAAGTACCAACATCATCCACCTATAAAACCTCTGCTATCTCATGTAGATGTGAACATAACTCACTGCTG
17630_62	C/T	1	CTGCAGAGCTGGAATCACACTTGGCCAACAGGAAGGATAAAACAGATAGTGTTCTTTAAAT[C/T]GGCTTTAATGGATAAAATCAATCCTTTTTGCCACAGCTTGCTTTCTCATAGTGCCTATTGTTATCTCTGCATACATACTTAGATCTATGCTGCATTTGTTGCTTTTAGATTTAGTGAAGTCAAGATGTAAGACCTCGT
17676_127	A/T	2	CTGCAGTTGTTTATAGTTGCAAAACTGCATGTAAGGGTAAGCTCCCTTTGAGTGGAGCTTCCTAAAATAATTTGGTGGTTGCTGCTGAGAATTTTTAGGAATATTCTGAAGGTGATCGTCCTATTA[A/T]GTCAGTGGTGCAGCTCCTTAAAAATTTAAGCTCTGAACTTTAGGGTAGATGGCGGTCTCATGAAGATTGCAGC
18371_155	T/A	2	CTGCAGCCTGCCCTAAAACGTGGTGACATTTTGAAAAAGAAAATGCTAATTTCAAAATTCAGCACTCTGTAGATAATTTTTAAAATTTCAGCTTCTATAAGTGTTCAAGTTATTTTAATAGCTAGCTCCCTGTTGTGCCCAAAATTCGTCGAAA[T/A]TTTTTGTTATCCAAAAACCAAATCTCCATATCCAAAAACTCACAG
18536_64	T/A	2	CTGCAGTCACTTCCCTCATTGAGCACACATACACATCAATAGTTTATAGACAATACAAAAAAT[T/A]AAGACTCGGTTGAAAATAATATTAACAAAACAAAAGGCCAGAAGAGATCTCACATATCCACACTAAATATCAGCCAAGTACCTGTAAGCTAGTTAATACTCTTGCTTTAAGGGTGTGATCCATGGTTAGCTCATGG
18825_68	A/G	1	CTGCAGATGAAAGCCATCCACAAATTCAATTGCTAACTTGCCTGCAAAACTTGTAATCCCTTGCCAA[A/G]ATTCCTTTTTTGAGATCCAAGAGCAAGGAGAGTCCTAAGATACTCGAAGGGAAGAGTTCCTATTTGAAATCTAAGAATCTGAGGAATCTTGTTCTAGATAAGCTGAGGGGTATTAAATGAAAAGATAGAGGA
18882_160	A/G	1	CTGCAGGGCAAAATACTTTAGCAATGCCTTGTAGACCTCTATACCTGCATACATATCCTTGGCTTCCATCTCTCTAATCAAAGACTCTCCTGCTTCAGGCATTCCTACCCTCACATAAGCCATTATCATTGCACCATATGCTTTTTGATCCAGTTGAAG[A/G]CCAGATAGTCTCATCTTATCAAATGTATGTCTTGCCTTAT
19160_119	T/G	2	CTGCAGGGTGGGGGGCGCCCTTACTTATAATAGTAGGGGGTTAATAATAGTTGGGCAACTATAATCCCCTTCATGGCCCTACCTAAAATTCTATGGATGGGCTATGGGCCTGCTAGAG[T/G]TGCCATTACTATGCCCCTGCACCACCCCCCCAATAATAAGTTGTTGAATAGTTAAGATACCTCCAACTATAATGAGAGGAG
19165_137	C/G	1	CTGCAGCTCTATCAGTTCCATATTTTCAAGACTGACCTAACAAGTAAAAGAAGAATGTGGGTGCTTCTTTTGTTTAAAACACATAAGATCTGTGCAGCTCTATTAGAGCCATATTTTCAAGATTGACCTTGCACAC[C/G]TTTACGACAAATCCTTCAAATTTAAAAATATCAAAACACATGATGGCAAGAATGGATGCAGGC
19553_85	G/A	2	CTGCAGGACACATGTCAGGGTCCTATCTCTGCCAAATTTCAAAGGGCTGAAGCTAAAGCCTTGCAATATAAACGTCTTTTTCAA[G/A]GAAAGTTATGGGGATTTGTGTTAAAGGAAAATGTTACCACACAGCAGAAGGCAGGCATTCGCTGTGGAAGGAATTAGTTAACTTTTCATGTGATGGAAAATCATTAAGAAAAAGT
19809_45	G/A	1	CTGCAGTCTATATTTATTCCTCTGGTAGGTGACAATAGTTATAC[G/A]TTCAAATTCAAATTCACATTCAACACTACTTTCAACATGATACATATTGCAAACTACAATGGTTATTCCAATCTATTCCCTTTCACCAAACTGTGCATAATTAGTCTCTTGGAAATGGACATTGGAGGGAAATTTGAAAAGCCAAAGTTCTTGTG
19942_106	T/A	2	CTGCAGATGATGTCTATCTGACTGTTGGCTGTCTACAAAGTATTGATTTTGCTATGAAAGTCTTAGGTCGTGAAGGTGCAAACATTCTTCTTCCAAGGCCAGGAT[T/A]TCCATATTATGACTCAATAGTGGCATACAAAGGTATTGAAGTCAGGTATTATGATTACTGCCAGAAAGAGATTGGGAAATTGACTTGGGTCAAG
20726_122	C/T	1	CTGCAGCATGATGTGCAGGAGCTGAACCGTGTGTTGTGTGAGAAACTAGAGGACAAAATGAAGGTCTTGAATTTTGTATTTAATCTGTGATTGTCCCAGCACCCCAATTTCTCATGAAGTT[C/T]TTGTTGAAATTGAAAGCTTCTTTATACTGTTATATTTCAGGGTACTGCTGTGGAGGGTACAATACAACAACTATTTGA
211_106	A/T	2	CTGCAGCATTGCATGTAAATAGGTGCATAGTAAAGCTTTTAGGAAAAGGTCAAGAAGATGTTCTAAAATGAGACTGAAAAACTACATAGAAAAAGGGTACCTGCG[A/T]GCTAATATGTCATTTTACTGTACAGAATGCGGGTCACTGATGGAGCTGGACTATCTGAGAAAGTTGACAGTTCATCAATCTAAATTCAACAAAA
21351_162	G/A	1	CTGCAGCCTAAGAAGAGGAAATCCAATGATAACAATGAAACCCAACTGTGTAGGGGATCCAGATGCACAAAATCAACTCATTTCCAATTTTTCACATATCAGATTTTCTAAGAAGAAACATTTTCACCTATCAAATCTTCTTCTGAGGATGAAGAAACACA[G/A]AACTATTCTATCTCATGTCGATTTCTCTGTCATGAATC
21440_152	T/C	1	CTGCAGGCATAATATACAGAATCCAAGAAAACTTTATAAGAAAAAGAATGGATGTTATCATGTTCTGTCTTATGGCAATTGATATGTTATAACAGCTAATCATGTCAACCACAAATCGCAAAGTTTATAATTTACTAGTGTCAGTAGATAA[T/C]GATTTCATAATAGGGAACAAATCAGTGGAGAAAGGGAAACATGTTCAG
21548_155	C/G	1	CTGCAGATGATCGCAGATTAGAGTACTTAATATAGAAGTATGCAGATTGCAGTAACTGGAGATAATAACATGTATTGTGGTGGATGCTTTCTCTCAATCACTGGATCAATATATATATATGAGAACAAAGGGTCATGTTAATGTAGAGACATGA[C/G]CCTACATGGCATGAGCCACATAGGGTCATGTTCCTACATCAACAT
21832_135	T/C	1	CTGCAGTGCAAGAAAACACCATGAGAAATAAACATTTTCAAAAATATTTATCGTTCTTTTCCCAAAATAGAAAACTATCTATGTGCTTTCAGGTGCTCTGATGTGCTACTCTCTAGGTGATTTATTTGCAACCA[T/C]ATGCTGACATATAAGAAATTTTGGTCCTTACATACAAAATATGATGTCAACATTCAGCTTCAAGC
22176_62	C/T	1	CTGCAGCTCGAGGGGACGGATCCATGGTGAGATTGCTGATGCAGAAAGGGGCTACGAAAGA[C/T]ATCAGGAACAAGCACGGGAAGACCTCATATGACCTTGCGGTGGACACAGGGGACTCCGCTTTGCTCGATTGCTGCGTCTCGGCGATGGCCTCCGTAGGGCTGCAAGGCGCGGGGATTTGCGAAGTCTGCAACGGTGCC
22479_130	T/C	2	CTGCAGAATGTCTACTACAGTTGGAGCCACAGAATGCTGGTATACGTGTGTTGCTTTCAAACATATATGCTGAAGCTGGCAGGTGGGATGATGTAAGAAAAGTGCGAAAAATGATGAAAGACACAAAGG[T/C]TGTAAAGGACCAGGACAAAGCTGGATTGAGATCAAGGAATCAGTGCATGTGTTCTATGCCGAAGACCAAT
22617_130	C/A	2	CTGCAGTCGATCTGCCTTGCCCACAAAAATGGCTGGTAGGATATGTAAAACAGCACCATAATGGCTTGTCGATCTAGCAATCTCAAATTGTGATCGGAAATCAATATCAACAATTAGCCTCTCCGACTT[C/A]CCCATCGGTCCCACTCCTTCCAGGATAACATCAATGTATTCATACTCTCCTGAAGGAAAATACAAATGTA
22877_36	G/A	2	CTGCAGATTGGGGTGGAACAGAAAAATGGTGATGT[G/A]CAGGAAACTAAATGGAGTATAAGTTCAAACCCTAACAGCCTATTCGTTGATGTAAATGAAAATAGTTCTCCCTGTACTCTGATACCAATGTCAGGCCAGGAAATTGGACTGCAAAGCAAAATTTGACATTGTATTGATGCAAAATCCAAGATGGGATTATTGTT
23028_86	G/T	1	CTGCAGTAATGGACAAAGACGCTATAGTCTTTCACTTATTCATTTAACTGGTTGCACATAAAATAAAGTAAATGCATAAGCTTTG[G/T]TGGATCTTTTGTTATCAATATCAATATCCCTATTTGCATGGTTTAATAATAAAGGATAATAAATTTAAAATACAAAAGAGTAATAATAAAATTTAAAATTAAAAGGGTAATATT
23735_62	T/G	2	CTGCAGATAATAAAAATACATATACACTGGGCATATATTTATAAACTCAACAACATAATAG[T/G]TTGAAAGATAGAGTTAACAGAAATCACAAATTAAAAGGTGTTATATATTTAAGACTTAGGGCAATATTAAGCAAACTGGTCAAACACAATAAATGGAGCAGTGATATGTATGATGAATCTATTAAAATAAGAAGGCCT
23845_33	T/C	2	CTGCAGTAGAAAGGAAAATCAGCACACCAATT[T/C]CAAAAGTATTTTAAGTGCAGATAAATATTCATATACATATTGATGTCTACACTAATGCCCACTTTTTATCTATATATTTCTTTCTTACAGAAAACGTAACCTAAACAATAATTTGATTGCAAATACTAAAATGGTAATAGCACCTAGGAGAACATAACAACACATGA
2390_145	C/T	2	CTGCAGACACTGAATGGCCATGGGATTGAGACCGGAAAGGGCTTGGCGTGCAAACTCGTCGTCTGTCTGCCACGCATGCTCGTCAGCTGCAAGACAGAAATAAAAGTTCAAATTTTATTGTACCAATGATAGGTTATTCGATAA[C/T]TAGTACTAGCATAAAACCGCTTGAAATTTATTTAAATGAATGGTGGTGATCTCGC
2715_44	A/C	2	CTGCAGTGTGAGTGAATGTTTTTAACCATCACCAACATACTAG[A/C]CAGATTATCATTGATAAGTTTTTCTTTTGTTTTAAACTATGTTTAAAATTTTTGGTCGATCTGTCTAAATTGTGTATTATTATCATGACGATTGTAATGTATGCCACTTAATTTTAAACGCCTACTTTTCATATGCTTTAAAAATATCACATTAAG
2815_174	G/A	2	CTGCAGTGCTTTTTCTGCAACATTTGCCCTTTTCGTTTCAATTGCAGTTCTACTGGTATCCCCTGTTTCTGCTGCCCTAATTTTCTCCCGCGCCCTGCTTCCCCACTCTTACATCTGACCGGATTTGCTCTCCAAATCCGTGAATTCGTCAATTCCGCCGGATTTCCGAATTC[G/A]CGCTACACGCATCTGATTTGCAGCAA
3099_117	T/A	2	CTGCAGTCATTAGAACCTATGCATTTCTGCAATAAGATACAAATCGCTAAAATAAGTGGCAATTGAAAAGCAGTTGAAAAATATGTTTTACTCTAGCTATTATGACGATCTGCAAT[T/A]TTACAGATATTGTGATGATATCACTGAAAGACAAAGAAAAATGGAATCCAGTAAAGGACATGCCTTTCATAGTTGAGGGCACA
3293_161	T/A	2	CTGCAGAACAAGTACTTTGAATATAGGCTCACATGCTCTATAATCCATGAAAGTCAGTATCGCAAGTGCCTGTATATAGGTAGAATTACGAAGTAAATGGGCTACTCTACGTGCTTTATTAGAAGACATACGTACATGCTTAGCTACAGCTCGTATTTTT[T/A]ATAGTTGAATTTAAATCTAAATCCCTATTTTGAAATATC
3946_69	A/G	1	CTGCAGCGCAGGCGATAGTCACTGAGCTTGGAATGTCCAATCCTAATGACAAGGGTGAAGTTGTAACA[A/G]AACACTTACTAACAACAATGAAACATCAAGTAGGTGGTCAGGACAATACATCATTGTTAACAACTATTCTGAAGAATGCTGGTATAATTGCAAAAGCCTTGGCCTCTTTGCTCTCTGGAATGGTCCAGGAC
4003_165	G/A	1	CTGCAGTTGGCACTAGGCTATCAATATTTTTGCTTGTATTAGAATACAAGCTTAATTTTTGAAAGATAGGGACCAGGCATGAATCTGTGGGATGAATGGCCCACGACAGAGGCTTTCACATTAAATTGTGTCAACCAAATCTAAAGCAATATTCTAAAAACCAT[G/A]CTCAACTCAAGCAAGGAAGTCATAAAAGAATCATT
4131_172	C/T	1	CTGCAGCTTTGGTACAAAACTAAAAGGCGAGAGATTTCTATTTCCTTTCATTGATAATTGATAAATCGTGATTAAATTTGTTTTTGATATGCGACCTATGGAAATTTAAGTCAATATAATAAACACTTTAATTTTTGAGAGAGTAATTACCTATGAAGGGATTGACTTTTT[C/T]TTAGTGTATGAATCATTTATCTATAACC
4227_95	A/C	1	CTGCAGAAATTTCGTAGAGCTAAAAAGGTATTAGACCTCGTTTATTTTTCCAATCTTTGAACGCGGGTAAATTGAGAATTTTCTTGTTCTTCCT[A/C]TTTTTCAATGCCCTCTGAAAACAAAACATGGCATGTTATGAAGAAATTAGCTGCAACTTTTTACCGCGCATGTACTTCTGAAACGAAGCAGCTCCAGTTCATTCC
4497_57	C/T	2	CTGCAGAGACCGTCAATAGCCTAAAGATTGTAAGAATAAATAGCATAAACCCTGCC[C/T]ACATTTCGTTTCTTTCCTTGCTGATCTATAACATGTTAGGTTTCACAATATTCTATTTAAACAATTTGAAATGCTGAGAGAAAAAGCCGAGAAGAATTAGCAGTTAGTTTGTGTTTCCCAGAAATTTGTTCTGTTCCTAAAAT
465_128	G/C	2	CTGCAGCCGGGGCTATATTCTTTAGCTTTTTGGTTAGCTTAGAACGGTTGCGAGTCATGATTACGTTGACAACGCGAGGCATGCTGGTGCATCGGAATAAACCCGGGCCCAGCAGTGGATGGGAAGG[G/C]CCACCCACTGCTTTATTGGCAATGCTGGGGTGGCCGGGTGGCGCACGAAATTTGAGCCGATTCTTCCGATCC
4913_26	C/A	2	CTGCAGATGTGAAATTATAGATACT[C/A]ACACGTTCACTTTGCCTAAGAATAATAATGCCACATTGATCTGTCTACATGCTCATACGGTCAATCTACTCTATGGATGTTATTGATCTGCTTATATTCCATTGGTACTATTACATGCACGACTGACTTACTGAATATAAAACAATCTGTTACAGGACAATTACAGTAAGATCA
5134_143	C/T	1	CTGCAGCATTCATGGGAACGTCTATGTCCAAAATACCCATGTGTTGTTTCAGAATGGCTCTGTGCTGAGTATTAAGAACTCACGTGATGGCTCCATCCAAAAAATGCCCCAATCGACTCCAATATGCTCATTCCTCCCTTTT[C/T]GTACTAGGGCATCAAGAAAGACATTCAACCCATGGACTTGTTTGTTTGAAGCTTTTT
5447_151	G/T	2	CTGCAGAAGCAGTAGGAAGAGGAAGGACCTCTCCCATTCCTTGCATTTATATGTTGTTGCTTTGATGCTTATGGTTGAAGTCAGAAAATTGTGTGAAAGTAGCTCTACCCAACTGCATTAGGCAAAGAGATCCAGTAACTGTTAGGATTA[G/T]GGGGGTAATGATCATGATAGAGTGTTGTCTTAGATACTTATAATAGCTG
5662_100	C/G	2	CTGCAGAGGACTCTCCTGCTAGTCAACGTAAGGATGGTCAGCTAAACAAATGAACCGGCTATAGCTATAAAAAATGCTTGAACTCAGCCTGAACTGCCC[C/G]GTAGGGCCGATGGGTGATTATGCAGAGGCCATAGAGATTATAGGCGATAGTTGTTTACTGTCCCCCTATATATTGAAGGGCTCGCCCTATATATTGAAGG
5822_134	G/A	2	CTGCAGAGTTATTTCCTCATTCAATCTTATCTTCTGCTCTTTATCCACTCCACTCTTTTTTTCTTACATATTCTCTGTATTCATTGTACTTCATTTTCTCATGAATGAATTCTCTCTTCCCTTTATATCATTC[G/A]TATTTTTCATGAACAATTGTGACTCTCTAAATAGACATCTCTTTTCAATATATATTACATCGCACC
5868_118	G/A	2	CTGCAGTCTGTTTGAGTCATTTACAGCCATCATCTTGCTGCCATTACACTGTACTAGAGCCATGATGTGTAGTGCAAGTTCCAATCAGTCCTGGGAGTAGCTAAATCAGGGTTCAGA[G/A]GCTCCACAAATGTTAGAGAAGCTCTGTAGCTGTTCTAGTGTTGCAACATCAATCATTATTTGGTATGTAAGAAATAAGACAC
644_98	G/A	2	CTGCAGGGCCATGATTGGCTAACAGACTGAAAGCGGGTGATATAGATGCAGAAATGCAACGTTCTTCTGGTTGCAACGATAGGCTTCCAGATGGATG[G/A]AAAAGTCCCACAATTGGATGGGATGGGATAGAAGAGGCGCCTAGTGGGGCTCCTTCTGATTCTGCCATAACGCATCTCTCCCCCCCTACTATTTTAAGGGCT
6837_73	G/T	2	CTGCAGGAAAATCATCCAAACAAAAAGGGGCTGTGTTCTATTTGGGTAAAATCATAGACAACCTAGATAAAA[G/T]GAGTATCTTTTAATTACTGGCATTTGCCATCTTTGAAATTTTGGCTTTGTGCTTATCTTTAAATGCTCCAAGATGGTAGATAGCTTGGTTGGACACTTAAATATATTGCTGTAATGTTGTCAATATA
6912_133	G/A	1	CTGCAGCATCATGCATGCTAAAGCGTTGCAGAATTTCGAAAGGCCTAAATGCAGGACAAAAGTTAGTTTCTACTTTATGCATTCCTACATAATTTCTTGGCAAATATGTAATCTTGAAGTATTAAGCAATCC[G/A]AATATAAGCCATGTTTGCGAATTTTACACAAGTGTACCAGTAGGAATATTAAAGAAAATAGGGGCTC
7145_33	T/C	1	CTGCAGTAGAAAGGAAAATCAGCACACCAATT[T/C]CAAAAGTATTTTTAAGTGCAGATAAATATTCATATACATATTGATGTCTACACTAATGCCCACTTTTTATCTATATATTTCTTTCTTACAGAAAACGTAACCTAAACAATAATTTGATTGCAAATACTAAAATGGTAATAGCACCTAGGAGAACATAACAACACATG
7147_80	C/T	1	CTGCAGATTCCCTTGGATCCACAAGTTATCAAGCACAGTGGATGGCACCACATTCGATATCCAACGATGAGGCCTTCCT[C/T]CCTATATATACTTTTCCTCCAAGAGGTTGGCCTCCTTCTAGAAGTCATAAAATAACATTTAATTTCAATTTCCAAAAGATTCACTTGTTTTCATAATAAATTGATTGCACTTTAAAGTAA
7411_88	A/T	1	CTGCAGTAAATTTTAAAAAAATGTAAATAAATTATCAGTAGCTTTAACACTTGATGGCAAAACGTGTTTAGGAAATACAATAATCGA[A/T]GGAAAGGGGCGTTTGGATCATGGATAGTGGGAGAGGATAAAAATTCCATTTCCATTTGTAACGTTGAAACGCGTTTTTCTTATGTTTGGAGTTATATTGATTATAAAAATGT
788_100	C/G	1	CTGCAGACTTGAGGTGTGGAGGGATTTTGTTAAGCAGTTTTGTGAATTTCATAATAAGGCTAGCCACTAATTCATTTTATTGCTTTTAAATGTTTGTGA[C/G]TTCATCTAGAACAATTTTTTGGGTCTCTGTGATCTCCATATTGTTCAACAAACATTCTTCTGAATTCAAGCCAGAAGCTGATTAGGGCATTTGGCAGATT
8375_147	A/T	2	CTGCAGAAAGAAAATGAAGTTGGAAATGGAATATATATAATTAGATTATAATTGGGTTCTAAAATATCTTTGGATGACATACTTTCGAACCTAGAAGAGCTGGATTTATCCAAGGTGGGCCAGTCTGATTGGAGATCCTTGAATTG[A/T]GCTTTGTAACCTCACTGGTCACCTTAGTCCAACACAGGATAATAGAGCACCTG
9164_153	G/A	1	CTGCAGATCAAGCATATAAATTTGAAAGAAAGGTAGTACAATGGATCCTATTTTCTTAACACTCTCAAGTCTGCTTTCTTTGGATAGACATCCTGTTTCTCGGTCCTCGCCAAAACATTCTCATCTCACCTTTATTAAATATCATTACAAAA[G/A]GAGGCGGCCTTTTACAAATAAGATGACTTTACTGTGAATGATGTCTC
9258_25	A/G	2	CTGCAGCATCTTGTGGTCCTCAAG[A/G]TCAATGAGGCAAATTGTGCACTCGAAGCCATTGAGCCCCTCCATAGATGTTCTTCATCAAGTTCCATCAAGGAGCTCATAAATCGCACCAATGATCAATAGTAATATATTCCTCTTCTGCTTTGATATTTGCCAAATCAAGAAGAGGTGCAATGATACAAGAGCATTATATTATG
9590_145	G/A	1	CTGCAGCACTTCCTTTCTGTTCGAAATTGAAAAAATGAATTATGGGTGAAGCTGCTTATTTTATTCCGAGTTTACCCTTTCAATAACCATTCGAAGGAAATCAATTAAGTCAAAACAGGTTGTAAATTTTGCAGCTAGAGCTGC[G/A]CAAAGAACTAGCTAGTAATAAGAAATAAAGCTTGTAGAAGAAAACAGACTGAGCT

**TABLE A2:** Primer details for all 6 SSR loci used in this study.

Loci	GenBank accession number	Repeat motif	Repeat length	Allele size range (BP)	Amplicon size	Forward primer sequence	Reverse primer sequence
8921 s	PQ700281	(TACA)	7	158–270	248	TTCACGCGAAACTCTCCAG	AATGGCTTAACTGCCTCTCG
8480 s	PQ700280	(TATG)	12	100–232	187	CCTGCAATCGATGTCTTCCG	TGCCAAATAGTAACACAGATGGG
576 s	PQ700277	(ATGT)	7	174–294	238	GTAGACTCACTCTCCTCGAATG	TCCAGCAATTGATCACAAGGG
9104 s	PQ700282	(ATGT)	8	156–220	152	GTGTTGCTCCCATCTAGCAG	CCTTACACCACTGGATGCTC
2346 s	PQ700278	(TACA)	7	234–258	229	AGGTCCCACACCACTTATCC	CACATGGTCTTCCTTGGAGTTG
JC035	PQ700279	(CA)	20	218–264	155	tgtgtttattctccccatct	cccccagttattctaaacatt

*Note:* All forward primers had the M13 sequence added to the 5′ end of the primer. JC035 was first described by Michalczyk et al. (2006). Loci 576s and 9104s were removed from final analyses for being null alleles.

**TABLE A3:** IDs (populations), whether an individual was removed, and genotypes of all individuals who shared genotypes in the SNP dataset.

Individual 1	Individual 2	Removed individual	Genotype
70 (CD)	86 (CD)	70	12/21/33/42/44/31/24/11/11/11/11/11/22/11/11/43/33/11/22/42/21/11/22/44/42/44/33/22/44/33/33/41/33/31/33/31/42/22/11/31/11
71 (CD)	83 (CD)	71	22/21/31/22/44/11/24/11/11/11/44/11/21/11/31/43/33/13/22/44/11/11/22/44/44/44/33/33/44/11/11/11/33/31/33/33/42/11/11/33/11
213 (*Nana*)	214 (*Nana*)	213	22/22/31/22/44/11/24/13/11/44/11/11/22/11/31/43/33/13/22/44/11/31/22/33/22/44/33/32/44/33/11/41/31/31/33/33/44/11/11/33/11
214 (*Nana*)	216 (*Nana*)	214	22/22/31/22/44/11/24/13/11/44/11/11/22/11/31/43/33/13/22/44/11/31/22/33/22/44/33/32/44/33/11/41/31/31/33/33/44/11/11/33/11
157 (*Hemi*)	158 (*Hemi*)	157	12/22/31/22/44/33/24/11/41/44/41/11/21/33/31/44/33/13/22/42/11/31/22/44/22/44/33/33/22/33/11/44/33/33/33/33/44/11/11/33/31
158 (*Hemi*)	159 (*Hemi*)	158	12/22/31/22/44/33/24/11/41/44/41/11/21/33/31/44/33/13/22/42/11/31/22/44/22/44/33/33/22/33/11/44/33/33/33/33/44/11/11/33/31
162 (*Hemi*)	167 (*Hemi*)	162	22/21/33/22/44/33/22/11/11/41/41/11/21/33/11/44/33/13/22/42/11/31/22/44/22/44/33/33/22/33/11/44/33/33/33/33/44/11/11/33/31

*Note:* Pairs of numbers are biallelic loci separated by dashes; numbers signify base pair: 1 = A, 2 = T, 3 = G, 4 = C. Only in those cases where individuals who shared a genotype and were from the same population was one of the clonal individuals removed.

**TABLE A4:** IDs (populations), whether an individual was removed, and genotypes of all individuals who shared genotypes in the SSR dataset.

Individual 1	Individual 2	Removed individual	Locus 1	Locus 2	Locus 3	Locus 4
22 (BG)	224 (*Nana*)	n/a	250/250	212/196	230/222	250/250
32 (BT)	280 (BF)	n/a	258/258	200/196	224/224	254/250
38 (BT)	228 (TF)	n/a	258/250	212/200	224/224	250/250
45 (BF)	15 (BG)	n/a	250/250	200/196	224/224	250/250
54 (BH)	27 (BG)	n/a	250/250	208/200	224/222	250/250
61 (CD)	79 (CD)	61	250/250	208/200	230/226	250/250
62 (CD)	84 (CD)	62	226/258	200/196	230/224	250/250
66 (CD)	83 (CD)	83	258/254	216/196	226/226	254/250
67 (CD)	85 (CD)	67	258/250	200/196	222/222	250/250
96 (CD)	210 (LM)	n/a	258/250	200/196	226/224	250/250
150 (FK)	181 (GA)	n/a	250/250	196/196	226/224	250/234
187 (*Hemi*)	193 (*Hemi*)	187	250/250	232/196	224/224	238/238
189 (*Hemi*)	192 (*Hemi*)	189	250/250	232/232	224/224	254/238
210 (LM)	211 (LM)	211	258/250	200/196	226/224	250/250
211 (LM)	313 (WW)	n/a	258/250	200/196	226/224	250/250
222 (LM)	226 (LM)	222	258/250	200/200	224/224	250/250
243 (*Nana*)	245 (*Nana*)	243	250/250	216/196	230/224	250/250
258 (PD)	261 (PD)	258	258/258	196/196	226/222	250/250

*Note:* Numbers are the number of base pairs at each locus, with alleles separated by a dash. Only in those cases where individuals who shared a genotype and were from the same population was one of the clonal individuals removed.
